# Electronic Fetal Monitoring–Prevention or Rescue?

**DOI:** 10.3389/fped.2020.00503

**Published:** 2020-08-27

**Authors:** Barry S. Schifrin

**Affiliations:** Department of Obstetrics & Gynecology, Western University of Health Sciences, Pomona, CA, United States

**Keywords:** EFM, classification of FHR patterns, fetal acidemia, obstetrical malpractice, defensive medicine, malpractice awards

## Introduction

This commentary represents a response to two recent contributions to the literature on electronic fetal monitoring (EFM) (1, 2). One article by Hirsch, raises concerns about the value of fetal monitoring in light of a very large judicial award of $50 million against an obstetrical service for a “brain-damaged baby” (1). In the other article entitled: “Fetal Heart Rate Monitoring: Still a Mystery More than Half a Century Later,” the authors present a Category II EFM tracing which creates for them uncertainty about whether the “fetal acid-base balance may be affected” and whether they may await spontaneous vaginal delivery or must consider more expedient operative delivery (2).

## Bewildering Fhr Patterns

The authors of the two articles share a bewilderment about the meaning of fetal heart rate (FHR) patterns and the problems of responding responsibly to them. They observe that despite the longevity of its implementation, the ubiquity of its use, and modifications to the classification of FHR patterns over the past 50 years, the interpretation of FHR patterns has continued to “befuddle obstetric care providers.”

In these responses, the authors are reflecting a broader bewilderment in the society of obstetricians. In the most recent coverage of the topic in Up-To-Date the author finds no unequivocal benefit to the use of EFM; further insisting that it is equivalent to intermittent auscultation (3). In a publication on the evaluation and response to Category II patterns, eighteen well-known authors confess that “As a medical community, we seem to know less than we thought we did 30 years ago regarding the utility of this ubiquitous technology.” They also aver that “Unfortunately, this body of work [EFM research] has primarily served to raise more questions than it has answered” (4). In a subsequent study their proposed scheme to manage Category II patterns was found to be of very limited benefit (5). One is reminded of Churchill's description of Russia as a “riddle wrapped in a mystery, inside an enigma.”

The “befuddlement” of obstetric care providers comes not only from the promulgation of what may be considered “defensive” classifications of FHR patterns (including—Categories I–III from ACOG), but also from a monolithic, and ill-considered view of the role of acidemia in the provenance of adverse fetal neurological outcome.

## The Classification of Fhr Patterns

The currently popular, three-category classification of fetal heart rate patterns in the United States (Category I–III) was introduced in 2008 without proper vetting and without attention to fundamental physiological principles (6). These comments may be applied to classifications from other national/regional organizations as well. These classifications, tied exclusively to the estimate of fetal acidemia and ensuing hypoxic-ischemic injury, attributes little importance to the proper assessment of uterine activity and ignores the concept of using the individual FHR pattern, over time, as its own control. Moreover, it imposes arbitrary definitions of tachycardia and bradycardia and takes no instruction from the recovery of the fetus from the individual deceleration or its evolution over time; there is no recognition of the importance of fetal behavior or the potential for the prospective identification of fetal neurological injury or intracranial hemorrhage (7, 8). They do not obey physiological principles (9).

Category II patterns offer disparate combinations of (1) decelerations (early, late or variable) with normal baseline features or (2) abnormal baseline features (altered variability, absent accelerations, tachycardia, bradycardia) without decelerations. Given the breadth of physiological and pathological conditions that may present with a Category II tracing or equivalent designation in other classifications (cord compression, head compression, placental insufficiency, medication effects, prematurity, fetal sleep cycles, existing injury, anomaly, etc.), it is unreasonable to consider that the metabolic status or the tissue oxygen reserve of each fetus, or the time to decompensation is the same. Indeed, “Category II” pattern may reflect a normal healthy fetus, but it does not exclude either fetal acidosis or fetal neurological injury (10–13).

Combining these disparate features and etiologies into a single classification and offering vague guidelines for their management including “continued surveillance and reevaluation” (3, 14) appears to have contributed to the “conundrum” for those providers trying to decide what the ubiquitous (identified in 70–80% of fetuses during labor) (15) Category II tracings mean, how to respond to them, it and how to counsel patients.

## Implementation of the Category System

The argument that the introduction of EFM was not accompanied by rigorous studies can also be made about the adoption of the various systems of classifying FHR patterns and tying it exclusively to the presence or absence of fetal acidemia. A greater deficiency was the failure to understand the provenance of intrapartum fetal injury based on the assessment of both immediate and long-term outcome, not just injury associated with a very low pH. Although ACOG guidelines, at least, accept the evolution of Category I to Category III as confirmation of an intrapartum injury, most babies injured during labor do not have severely pathological patterns (11), nor are they acidemic at birth. The de rigueur requirement of acidosis to make the correlation between fetal heart rate patterns and injury made a virtue out of necessity in that no measurements of greater relevance, such as fetal blood pressure or cerebral perfusion, were available.

Why is EFM not beneficial? It is universally agreed that fetal heart rate patterns reliably detect fetal hypoxia and are strongly related to adverse outcomes (16, 17). If the test of its value, however, rests with the correlation with pH or base deficit (BD) at the time of birth and not with long-term outcome, then the wrong question is being asked. On the other hand, if EFM has no preventive value, except to increase the cesarean section rate, what can possibly be the justification for either it or intermittent auscultation?

One can only agree with the enlightened notion of Andrews and Tivo that “measures should be employed in an effort to convert the category II to a category I tracing” (2). In this recommendation lies the likely reengineering of the approach to EFM as an instrument of preventive care rather than one geared to rescuing the fetus from an obviously hostile, presumably acidemic, environment (18). In this respect, it is necessary to place monitoring in its proper context by a careful evaluation of maternal, fetal and obstetrical risk factors. It is especially necessary to ensure adequate fetal reserve at the outset of monitoring; to scrupulously avoid excessive uterine activity (an independent risk factor for adverse outcome) irrespective of heart rate pattern and to titrate the mother's expulsive efforts according to the response of the fetus. Expulsive efforts dramatically increase further the intrauterine pressure and the distortion (molding) of the fetal head with potential compromise of fetal cerebral blood flow. These should be considered as primary instruments to prevent or improve abnormal FHR patterns and minimize the need for urgent intervention. Further, these initiatives must be taken as early as possible, and assessed for trajectory with each contraction. Failure to observe improvement after a reasonable number of contractions, not time, gives credence to the notion of a fetus on a trajectory of decreasing fetal reserve, irrespective of whether some specific pH or base BD value has been reached. There appears to be no clinical virtue to seeing how close one comes to catastrophe before intervening (rescuing). Indeed, withholding intervention until the pattern reaches Category III makes the determination of fetal acidemia more important than a normal fetal outcome.

## Malpractice Allegations

Finally, we come to the proverbial elephant in the room—the allegation of obstetrical malpractice that rests with the interpretation of the EFM tracing—a concern so ubiquitous that it appears in many if not most articles on fetal monitoring. This is not without cause; the substandard response to FHR patterns is a conspicuous mainstay of preventable injury worldwide whether the tribunal is the courtroom or organizational review (19, 20).

In his article, Hirsch provides neither the tracing nor sufficient information to evaluate its role in the outcome of the patient or the medico-legal encounter. We are also not informed of the positions of the attorneys for either side or most critically, the credibility of the various witnesses including the experts and the defendant. One conclusion that can be drawn without knowing any of these details, however, is that the jury was dismayed by the deportment of the defense.

The $50 million award that “hinged on the disputed interpretation of the fetal heart rate pattern,” was certainly an unusually large award and well above the “average” $1 million estimated average lifetime costs of health care, including costs for productivity, and for social outlays (Morbidity and Mortality Weekly Report-MMWR). These estimates do not include out of pocket expenses, lost wages, emergency room visits, over-the-counter medications, caregiving expenses, among others which are greater than average if the infant with severe brain-damage has a close to normal life expectancy. Nor does it include the toll paid by the participants on both sides of the often lengthy, often dispiriting, process of discovery that characterizes Western litigation. Nevertheless, the questions must be asked: what fact pattern could have so impelled the jury not only to a verdict of negligence, but to such a very large award? And does this not represent a breakdown of the legal system—a lottery? Was it the high-flown oratory of the plaintiff's expert? Andrews and Tivo allege that “only individuals with *post-hoc* knowledge of the neonatal outcome (plaintiff experts?) seem to be proficient at interpretation” (2). Was it the severity of the injury? Was the afflicted child brought into court to prey on the sympathies of the jurors? My own experience suggests that it is not negligence alone that accounts for such rare, “runaway” awards, but deception by the defense; by distorting the evidence about the value of both EFM and hands-on obstetrical care as vouchsafed by “authoritative” sources. The jury has no other mechanism for penalizing the defense team—it has no option to say: “We award the plaintiff a fraction of the amount that we were considering and perhaps he/she will need, but we also want to show how disappointed we were by the actions of the defense that diminished our notion of the honor of the medical profession.”

Consider the following heart-felt testimony of an empathetic, defendant physician: “I am so sorry about this outcome; I grieve for the child and for the parents. I have never intended to harm anyone, much less a patient. I believed, then and now, that I was acting reasonably under the circumstances. Both for the patient's sake and my own, I wish we had the day to do over again.” Imagine the response of the very human jurors to this confession, not of medical error, but of human value, even of fallibility. Even in the dock (the witness stand) the doctor was trying to heal. Sometimes substandard care harms babies and mothers and is deserving of honest reckoning and adequate compensation. An award, however generous, can perhaps palliate the injury or perhaps lessen the heartache, but it is the humanity of the defendant that not only prevents “runaway” verdicts, but most importantly, also offers some solace (healing) to the parents while holding open the option of learning something from the experience that will benefit a future patient.

## Conclusion

Obstetrical health care providers continue to look for guidance in the poorly conceived classifications of FHR patterns largely unrelated to our understanding of fetal-maternal physiology and predicated on the notion of EFM as an instrument of rescue from “threatening” acidemia. We should acknowledge that these constructs and the “vagueness” of the management guidelines better protect the physician from the allegation of malpractice than the fetus from the potentially harmful stresses of labor and delivery. As the emotions present in these two contemporaneous articles clearly convey, we are all paying a dear price for that approach.

The quotation from Churchill cited above ends with: “but perhaps there is a key.” The “key” is to have a better understanding of the language (physiology) and trends of fetal heart rate patterns and to broadly adopt a less defensive posture that reorients our priorities so that we are more offended by bad outcomes than the specter of malpractice litigation. We must increase our support for parents before, during and after pregnancy and embrace the notion that what we do as obstetrical care providers does matter, perhaps long after the pregnancy is over. Health care providers also need support, but the classification of FHR patterns cannot immunize us against accountability or empathy.

## Author Contributions

BS conceived, wrote, and approved the final version of the manuscript.

## Conflict of Interest

The author declares that the research was conducted in the absence of any commercial or financial relationships that could be construed as a potential conflict of interest.
